# The Diverse Gait Dataset: Gait Segmentation Using Inertial Sensors for Pedestrian Localization with Different Genders, Heights and Walking Speeds

**DOI:** 10.3390/s22041678

**Published:** 2022-02-21

**Authors:** Chao Huang, Fuping Zhang, Zhengyi Xu, Jianming Wei

**Affiliations:** 1 Shanghai Advanced Research Institute, Chinese Academy of Sciences, Shanghai 201210, China; huangc@sari.ac.cn (C.H.); zhangfp@sari.ac.cn (F.Z.); wjm@sari.ac.cn (J.W.); 2 School of Electronic, Electrical and Communication Engineering, University of Chinese Academy of Sciences, Beijing 100049, China

**Keywords:** inertial measurement units, indoor localization, stride estimation, stride segmentation, gait recognition

## Abstract

Stride length estimation is one of the most crucial aspects of Pedestrian Dead Reckoning (PDR). Due to the measurement noise of inertial sensors, individual variances of pedestrians, and the uncertainty in pedestrians walking, there is a substantial error in the assessment of stride length, which causes the accumulated deviation of Pedestrian Dead Reckoning (PDR). With the help of multi-gait analysis, which decomposes strides in time and space with greater detail and accuracy, a novel and revolutionary stride estimating model or scheme could improve the performance of PDR on different users. This paper presents a diverse stride gait dataset by using inertial sensors that collect foot movement data from people of different genders, heights, and walking speeds. The dataset contains 4690 walking strides data and 19,083 gait labels. Based on the dataset, we propose a threshold-independent stride segmentation algorithm called SDATW and achieve an F-measure of 0.835. We also provide the detailed results of recognizing four gaits under different walking speeds, demonstrating the utility of our dataset for helping train stride segmentation algorithms and gait detection algorithms.

## 1. Introduction

Pedestrian localization is commonly used in maneuvers, fire drills, and mine rescues. Unlike GPS, optical, audio, and other sensor data, inertial measurements are infrastructure-independent, allowing them to be used for a terminal location in complex contexts [1,2]. As a result of the development of Micro-Electro-Mechanical Systems (MEMS), Inertial Measurement Units (IMUs) have become lightweight, low in power consumption, low cost, and non-intrusive to users, which are suitable characteristics for clinical and residential applications. Thus, IMU-based Pedestrian Dead Reckoning (PDR) has become popular and received considerable attention [3,4,5,6,7].

Stride length estimation, direction estimation, and position update are three key processes of PDR [8,9,10]. One of the most basic components is estimating stride length [11,12]. There are mainly two classes of approaches: the first kind of method is based on the integration of the accelerations, and the other techniques utilize various models to predict the stride length. The models can be further divided according to whether they are based on physical or statistical models. The double integration of acceleration in the forward direction is the most direct method for estimating stride length because it needs no assumption or user customization. However, it is not easy to obtain the forward acceleration from IMU measurements since each part of the body moves in different directions during walking. Biomechanical models for step length estimating, such as inverted pendulum models, are defined mainly by simplifying and approximating the mechanical movements of the human body. Nevertheless, due to the significant variability of pedestrians, these models need to be calibrated for each user. Mechanical models are also impacted by the non-negligible bias and noise of the IMU, which makes the distance error grow cubically over time or distance [13]. In order to reduce the cumulative error, Zero-Velocity-Update (ZUPT) was introduced to reset the integral computations for distance when the foot was recognized as remaining stationary on the ground [14,15,16]. Thanks to the periodicity of human gait, various statistical variables show a clear correlation with step length and can therefore work as features or predictors in statistical models. This type of method needs to create an empirical regression model based on the movement features of the pedestrian’s pelvis, feet, or legs and then fit the model parameters by utilizing the existing dataset to estimate the step length for walking. Li’s model demonstrates a linear relationship between step length and walking frequency [17]. Weinberg’s model utilizes the difference between the maximum and the minimum in vertical acceleration data within a step [18]. Kim’s model is only based on the mean acceleration within a step [19]. Scarlett’s model uses minimum, maximum and average acceleration to estimate step length [20]. Since the variability of individuals’ walking habits stems from gender, height, age, and walking speed, these empirical models require parameter customization for individual pedestrians. If the predicted data differ much from the training data distribution, then the accuracy of the statistical models will be low. In recent years, neural networks have been developed as a promising trend in the pedestrian localization area and have also been used for step length prediction [21,22]. They achieve better prediction accuracy than empirical models with the cost of larger-scale datasets and more massive computation consumption. With limited training data, neural networks are prone to be overfitted, and the requirement for a large number of computational resources prevents them from being used in wearable devices and embedded systems.

To summarize the preceding methods of stride length estimation approaches, they all treat a single stride as a whole processing item rather than dealing with more detailed decomposition and analysis. Firstly, a segment of signal corresponding to a stride must be detected, and then selected features need to be calculated and input into a pretrained model to derive a prediction of movement distance. However, stride length estimation is a case of a black box problem, and researchers are currently unable to investigate the effect of individual differences on stride length estimation. In the field of kinesiology, IMU-based mobile gait analysis enables a continuous and detailed insight into the motor performance of foot movements in multiple gait patterns, under more natural and realistic conditions compared to laboratory settings [23]. With the basis of estimating temporal and spatial parameters within a stride, gait analysis is not only used for the detection of stroke and Parkinson’s symptoms, but also for posture stability in the rehabilitation phase of treatment after injury [24,25,26,27,28]. Inspired by this, we think that it is possible to improve the accuracy of stride length estimation on the basis of gait analysis. By accurately dividing a stride into several gait segments, stride length estimation can be transformed into a fusion of several predictions from different gait analyses. However, there is still a lack of studies in the field of localization based on the analysis of gait signals as well as gait information and step length estimation for PDR application.

IMU-based gait recognition can be investigated on the basis of precise labels characterizing the semantic information corresponding to a specific segment of IMU data, and various datasets have been published [29]. These datasets provide cyclic data collected on different parts of the body by various kinds of sensors and are briefly described in Table 1. The Digital Biobank [8,10] and Sensor-based Gait Analysis Validation Data [30] mainly collected data from healthy elderly controls, PD patients, and geriatric patients by using an optical motion capture system to provide reference data, but the system must be built in a laboratory and has a limited scope. MAREA [31] was used to detect key movements in the gait, such as the heel touching the bottom and the toe off the ground. It provided gait data from 20 subjects, containing data of pressure sensors located on the soles of the feet and data of IMU sensors recording ankle and wrist movement. Each stride was divided into two phases, where the foot swings in the air and makes contact with the ground.

MAREA also provided foot movement data on a treadmill with changing speeds and inclination angles to help evaluate gait events detection, since the lower body movement kinetics of walking on a treadmill are similar to that of walking on the ground. However, the work in [34] found that the shear forces caused by the belts sliding over the treadmill significantly reduced propulsive force during late stance, so there exists considerable differences in gait variables between overground and treadmill walking. The Smart Annotation Cyclic Activities Dataset [32] provides foot movement recordings with camera frames as reference information. It provides stride borders in the foot movement sequence but with gait phases not mentioned. To summarize, thanks to the existing stride databases or gait datasets mentioned above, a large number of investigations into the biomechanics of foot movements have been made possible. However, there is still a lack of stride gait datasets providing both stride borders and gait phases from healthy subjects walking in overground with diverse parameters, such as gender, height, and walking speeds, which significantly affect gait variables in cyclic movement. Among the datasets that collect data from healthy pedestrians, the number of healthy subjects in our dataset is much greater than that of the sensor-based Gait Analysis Validation Data [27] and is comparable to the other two datasets [31,32]. Compared with MAREA [31], we offer more gait labels and foot movement data that are closer to the real application scenarios. Our dataset shares the same type of reference information as the Smart Annotation Cyclic Activities Dataset [32], but we provide reference data with a higher sampling frequency and more detailed gait labels.

One of the initial steps in most wearable gait analysis systems is to segment strides from continuous sensor data, which is a crucial component of the underlying signal processing pipeline. Various methods have been successfully applied to stride segmentation. By identifying peaks or valleys in the data sequence as significant and typical gait events, Weinberg suggested stride borders be recognized, and later dynamic threshold schemes were added to improve the accuracy [18,35,36]. Among the existing IMU-based adaptive stride segmentation methods, [37] proposed a technique which utilized an autocorrelation procedure to fine-tune the threshold for stride boundaries. However, unbiased autocorrelation estimates rely heavily on extracting meaningful information from the signal’s autocorrelation coefficients, necessitating a larger number of observed samples and thus lagged parameter values. The number of samples needed for analysis was said to be at least 400, but the sampling rate of current IMU modules is from 50 to 200 Hz, implying a lag of at least 2 s. As a result, parameter adjustments would be significantly delayed regarding the recognized stride.

In the study of [38], an adaptive procedure was added to the finite state machine algorithm in which six transition rules are empirically set for separating a full stride into six stages. The assumption behind these fixed state transfer criteria is that all strides are made up of a combination of six complete phases sequentially. The signal’s amplitude threshold, the signal’s derivative threshold, and the signal’s variance threshold were adjusted in the state machine based on sixty percent of the average of the local maxima and minima detected in the last three strides. If the recognized strides were less than three steps, the preset thresholds would be used. The method of parameter updating was based on the authors’ assumption that the signal distribution pattern of pedestrian walking follows a similar conditional pattern that transforms between distinct states within a certain range of speed fluctuations. However, the assumption of the cycle heel-strike, foot flat, middle mid-swing, heel off, toe-off, and middle mid-swing in the gait pattern limits the analysis for that particular forward walking gait. Although it is the most frequent phase sequence, it must be considered that some strides may omit one or more phases due to the uncertainty of pedestrian foot movements.

In the research of [39], a parameter adjustment mechanism was proposed to be added to the Hidden Markov Model to improve the adaptability of stride segmentation. Rather than analyzing the statistical characteristics of acceleration or gyroscope data, the system begins by calculating the Impulse Response Function (IRF) for the gyroscope signals at different stages to reflect the periodic impedance of the force at the ankle joints of the humans and robots. Additionally, it then compares the inverse of the Euclidean distance with the IRF of each stage at each moment. Similar to [38], the system assumed that the gait stages are fixed in mid-stance, terminal stance, swing, and loading response. The IRF of the current signal should be close to one of the four gait templates as the current estimated stride phase. The authors did not provide test results for this method on different subjects, so the robustness of the threshold adaptation for stride segmentation or gait recognition is unknown.

The research in [13] offered a positive correlation between the amplitude of the peak acceleration point within the heel strike phase and the threshold required to identify the zero-velocity phase and developed a regression model. The model was used to estimate the threshold value for the forthcoming zero-velocity phase based on the magnitude of the newly formed acceleration peak point. The utility of adaptive thresholds for zero-velocity phase detection, i.e., improving stride recognition accuracy, was only verified on a 75 m test-path with different walking and running speeds, while the cross-individual adaptation was not mentioned in the validation experiments of this approach. We infer that the empirical model parameters might need customization to each individual due to the influence of gender, height, and age on pedestrian gait patterns.

In summary, current adaptive stride segmentation methods are unable to recognize stride in cross-individual and wide speed domain scenarios with robustness due to the lag of the regulation mechanism, the fixation of the number of gait phases and complex transition conditions, and thr customization of threshold estimation models for different individuals.

Alternatively, template matching algorithms such as Dynamic Time Warping (DTW) were introduced for stride segmentation. DTW methods calculate the similarity between the input sequence and the template, which makes it well adapted to varying segment lengths and trivial distortion [8,40,41]. The template matching method, like standard DTW, can still distinguish the strides from signal series, referencing a standard stride template. The SDATW introduced in this research is trained on such a diverse gait dataset to maintain accuracy and consistency in cross-individual and broad speed domain scenarios.

From the perspective of frequency signal decomposition, wavelet analysis provided insight into determining stride borders, and it is suggested that better performance could be achieved in the frequency domain than in the time domain [42,43,44,45]. Another kind of method employs Hidden Markov Models (HMM) [46,47,48], residual neural networks [29], etc. These methods could achieve better detection accuracy in stride segmentation or gait recognition assignments with the assurance that large-scale training data should be offered and massive computing resources supplied. However, this requirement runs counter to the low-power-consumption nature of wearable device platforms.

The purpose of this paper consists of the following four parts. Firstly, we give a diverse gait dataset including IMU data and camera recordings of foot movement, which features a wide coverage of gender, height and walking speed. Secondly, we propose to divide a normal walking stride into four gait phases on the basis of biomechanics and annotate the whole dataset with four different gait labels. Last but not least, we offer a threshold-independent stride segmentation algorithm that requires no customization and is able to perform with adequate accuracy and robustness at different speeds. We take it a step further by evaluating gait recognition to provide a baseline for accurate gait analysis.

## 2. Materials and Methods

### 2.1. Subjects and Measurement Protocols

A total of 22 healthy volunteers (13 males, 9 females, age 32.5 ± 7.5 years) participated in the study and were divided into different groups according to gender and height information, as shown in Table 2. Each subject walked at three kinds of self-selected speeds along an indoor corridor of 46 m. The data collection area was free of obstacles and long enough, so they were asked to walk as usual while approximately keeping straight along the brick line between the floor tiles. We added this restriction with the consideration that using the brick line as an auxiliary beacon can help subjects maintain a straight walking direction. It is not only important for subjects that they can focus their attention on controlling the walking speed evenly and steadily but for the videographer that they could capture the full process of foot movement by camera. Although this limitation probably prevented us from fully simulating the walking state of a pedestrian in real application scenarios, we extended the diversity of the acquisition samples with three different walking speed gears. We let the subjects choose their own walking speed for three different daily scenarios: normal walking in the street, slower than normal choice such as thinking while walking, and faster than normal choice such as weaving through the crowds at a brisk pace to catch the upcoming bus. Furthermore, we chose a corridor that was 46 m long. The distance ensures that we collect a sufficient amount of data on pedestrians walking on a daily basis, since subjects are able to quickly adapt to this experimental environment built on a realistic scenario. By collecting walking data from 3 different speed gears and with 2 trails for each speed order, we believe such an acquisition scheme is able to capture the walking characteristics of the subject and essentially encompasses the regular and extreme states of that individual’s foot movement in daily life. By pooling pedestrian data across gender and height attributes, we can obtain a dataset that incorporates individual pedestrian differences and a wide range of stride gait states; hence, we call it the Diverse Gait Dataset.

### 2.2. Sensor System and Setup

**Figure 1:**
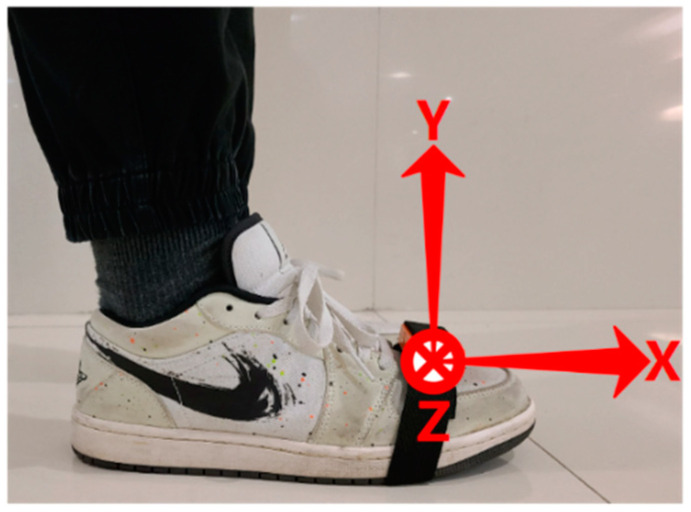
The IMU position and the direction of each axis.

Each subject wore an Xsens MTw IMU, which consists of a 3-axis accelerometer ($\pm 160\;m/s^{2}$), 3-axis gyroscope (±1200 deg/s) and a 3-axis magnetometer. The measurement module was attached to the in-step on the right side of the shoe with elastic bands and was connected via Bluetooth to the data acquisition software “MT Manager”. Figure 1 displays that position and orientation of the IMU in each trail of data collection. The accelerometer X and Y axes were pointing to the forward and upward direction, respectively, and the Z axis was pointing in the left direction. We used three different cellphones in different collection batches: an iPhone X (60fps, 720p), a HuaWei Mate 30pro (120fps, 1080p) and a One Plus 7pro (240fps, 1080p). Thanks to the smaller size and weight of the phone compared to traditional cameras, we were able to attach the phone to a tennis bat and just make sure that the fixation was secure. Cell phones’ video recording is not only able to overcome the impacts of unstable light in the field, but also, with its powerful anti-shake function and strong focus tracking ability, it can lock and track the foot as a target as long as we set the focus point on the camera. With the above guarantees, we can use the rear camera on the phone to record the foot movements through the grids between the tennis rackets with no obstruction. While the pedestrian was walking, the videographer needed to adjust the distance to the subject’s foot and angle of the racket so that all the foot movements were recorded in the view of camera.

### 2.3. Sensor Signals and Time Synchronization

**Figure 2:**
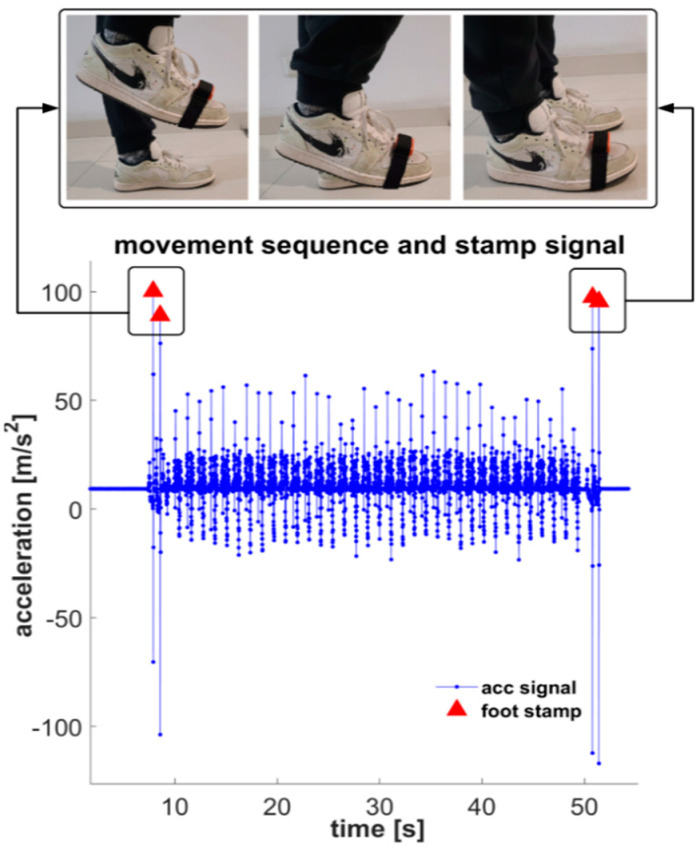
The acceleration magnitude of stomps is distinctly larger than the that of walking.

Each set of raw data consists of two parts: inertial motion data recorded by the IMU and foot motion images recorded by the phone camera. Since the IMU does not have a triggering mechanism for data acquisition, we offer a scheme to synchronize the IMU data and image frames after data collection. At the beginning of each trail of data acquisition, we asked the subjects to follow a pre-designed action: before starting to walk and after walking to the end point and standing firmly, the single foot wearing the IMU should stamp the ground vertically and swiftly [49]. This action is distinctly different from the foot movement during walking and can be reliably and accurately identified in the IMU data and video as shown in Figure 2. Based on the timestamp of the stomp actions, we can calculate the time offset that the IMU data lags behind the video stream. Then, we manually delayed the timestamp of image frames in the timeline for the period of offset value. It can be verified in the experiments that the time differences between the last three stamps and the corresponding image frames are almost zero. With this guarantee and consistent frequency in the video stream and IMU sequence, we could align the IMU data with the gait labels obtained from the images and maintain very high time accuracy in the annotation process.

### 2.4. Manual Data Labeling

The following section describes the concepts of manual gait division and the process of data annotation.

#### 2.4.1. Gait Modes

**Figure 3:**
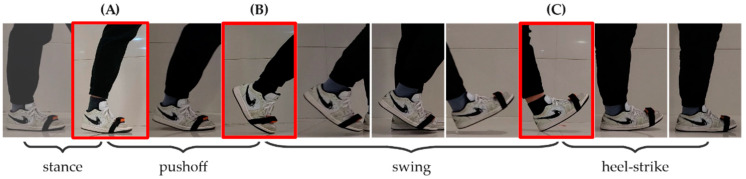
The movement of a whole stride can be divided into four gait phases. (**A**) displays that the heel just leaves the ground at the end of stance phase; (**B**) displays that the toe is going to leave the ground at the end of pushoff phase; (**C**) displays that the heel touches the ground at the end of swing phase.

When humans are walking, the body relies on the support of the feet to maintain the balance of the torso between travels. The torso generates forward or backward momentum with the ground’s reaction force on the feet to achieve startup and cushioning. Research in the field of human motor health defines a pedestrian stride as the process of a foot leaving the ground until it leaves the ground again. Based on biomechanics, this process could be divided into four different gait patterns shown in Figure 3: (1) pushoff phase: the ankle exerts force to make the heel and palm leave the ground sequentially; (2) swing phase: after the toes leave the ground, the foot begins swinging in the direction of travel in a pendulum-like motion; (3) heel-strike phase: the heel firstly comes into contact with the ground and takes the ground impact, and then the foot gradually makes contact with the ground to transfer the ground force to the arch to alleviate the impact; (4) stance phase: in this phase, the foot remains relatively stationary on the ground and supports the center of gravity of the torso, maintaining the body’s balance and preparing for the next gait phase, i.e., the push phase. These four gait patterns constitute a continuous cycle corresponding to the periodic motion of a single foot during walking. Based on the above discussion, the video frames can be utilized to generate four labels in our dataset: “pushoff, swing, heelstrike, and stance” corresponding to four different gait patterns, respectively.

#### 2.4.2. Data Annotation

Since the transitions between gaits are made in chronological order according to a fixed tight relationship, when dividing the gaits in the video stream, we do so by manually capturing the key movements of the feet in the gaits.

The process of producing labels is based on ELAN software, which was released in the year 2000 by the Max Planck Institute for Psycholinguistics in the Netherlands to label audio signals. It supports labeling of audio, video and audio-video multi-streaming data and is recognized as professional labeling software in psychology, medicine, psychiatry, education and behavioral research [50]. ELAN defaults to a semantic layer associated with the index position of the data to be labeled, then generates a label file of the data by placing the words entered by the user in the semantic layer.

**Figure 4:**
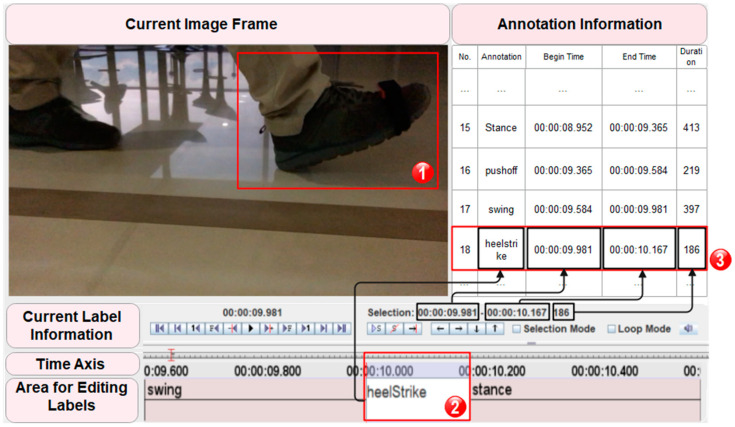
In the ELAN interface, the Current Image Frame shows the target’s movement state at the moment; the Current Label Information shows the time range corresponding to the series of images selected by the user. When a specific movement state is captured (box 1), the user stops opening the next frame and edits the label for the corresponding gait in the Area for Editing Labels (box 2); Annotation Information records the detailed information that has been labeled so far, and the latest label item is located in the bottom of label history (box 3).

We can obtain each image frame of foot motion and its corresponding timestamp in ELAN. By manually identifying and picking the image frames corresponding to different significant foot movements, we annotated all the foot motion data involved in this dataset and generated label files. It should be noted that manual annotation is a very time-consuming task. We could have taken the alternative of using existing gait recognition methods, such as Hidden Markov Models [46], but the parameters of the model or detection algorithms still require fine-tuning before real application. Additionally, in the case of unknown generalizability of methods, the detection results will require manual verification inevitably. Therefore, we sticked to this conventional approach to generate reliable and accurate labeling information. The flow of data annotation is shown in Figure 4.

#### 2.4.3. Data File Description

The data set consists of the following types of files: IMU data files, ELAN exported label files, subject information (height, gender, walking speed type, etc.). The IMU data includes the following: time_stamp_0, time_stamp_1, acc_x, acc_y, acc_z, turnrate_x, turnrate_y, turnrate_z, magnetometer_x, magnetometer_x, magnetometer_y, magnetometer_z. In that order, the two timestamp readings, tri-axis acceleration data, tri-axis gyroscope data and tri-axis magnetometer data are represented. The timestamp readings are recorded in sample order, which corresponds to 0.01 s, the acceleration data are in units of $m/s^{2}$, the angular velocity data are in units of rad/s, and the magnetometer readings are normalized to the Earth’s magnetic field strength. The tag file is suffixed with “.eaf”, where all labeled words are encoded and each code corresponds to the respective timestamp.

### 2.5. Stride Segmentation Method

In this part of the work, we chose the DTW method for stride segmentation because it shows satisfactory adaptability to different time lengths and varying signal amplitudes. We reproduced the multi-dimensional subsequence Dynamic Time Warping (msDTW) method and validated its performance in the diverse gait dataset. We refer interested readers to [51] for computation details of msDTW. However, we found that the msDTW works well on the basis of a grid search scheme to select a specific threshold value that is utilized when searching for the stride borders. If a valley point in the accumulated distance between the template signal and the input sequence is less than the threshold, then the segment between the last valley point that meets the condition and the newly detected valley point will be regarded as a new stride. If the threshold is too large, then pseudo-strides will be detected; if the threshold is too small, then normal strides are prone to being missed. In order to make the stride segmentation process independent of the threshold, we propose a novel method, SDATW, based on IMU-subsequence-shape-descriptors and the augmented time warping process. The following section describes the process of our algorithm.

#### 2.5.1. Template Generation

Stride template is defined as $B={\left\{\beta_{0},\beta_{1},\ldots ,\beta_{n-1}\right\}}^{T},\;B\in R^{n},\;\beta_{i}\in R^{6}$, where each sample in template consists of the item from 3-axis accelerometer and 3-axis gyroscope. We evenly select 30 percent of the manually labeled stride segments from each height group, then each axis in every segment was interpolated or down-sampled to a length of 200 samples [8]. Consequently, we get a stride-database with full-speed-range coverage for each height group. Finally, all the strides in the database were averaged sample by sample to generate a template representative of a compromise for a wide speed range and individual differences in strides.

#### 2.5.2. Data Normalization

In this literature, we use z-normalization, which is a necessity for accuracy and generalization of DTW methods [52]. Z-normalization refers to the process of normalizing every sample in a series of data such that the mean of all values is 0 and the standard deviation is 1, so it helps to reduce the impact of outlier points on recognition results. The calculation is shown in the following formula:

(1) $$acc_{norm,i}=\frac{acc_{i}-\bar{acc}}{S}\;,\;i=1,2,\ldots ,n$$

where $\bar{acc}$ is the mean value of the accelerometer series, and S is the unbiased estimation of the standard deviation:

(2) $$S=\frac{1}{n-1}\;\sum_{i=1}^{n}{\left(acc_{i}-\bar{acc}\right)}^{2}$$

Since only normalized data were used in further calculation, the index norm is omitted for simplicity.

#### 2.5.3. Calculation of Distance Matrix of Shape Descriptor Sequence

Intuitively, the DTW algorithm searches for the minimum cumulative distance when matching sample points by measuring the similarity of two time series of different lengths. We formally design the query sequences $A={\left\{\alpha_{0},\alpha_{1},\ldots ,\alpha_{m-1}\right\}}^{T},\;A\in R^{m}$ and template $B={\left\{\beta_{0},\beta_{1},\ldots ,\beta_{n-1}\right\}}^{T},\;B\in R^{n}$. We create and initialize the distance accumulation matrix $D_{m+1,n+1}:$

(3) $$D\left(i,j\right)=\{\begin{array}{cc} 0, & i=\left\{0,1,\ldots ,m-1\right\},j=0\\ 0, & i=0,j=\left\{0,1,\ldots ,n-1\right\}\\ inf, & else \end{array}$$

To calculate the time warping distance matrix between the sample points of the sequence, conventionally the elements of $D_{m+1,n+1}$ are calculated as:

(4) D(i,j)=dist(i,j)+min{D(i−1,j),D(i,j−1),D(i−1,j−1)} 

**Figure 5:**
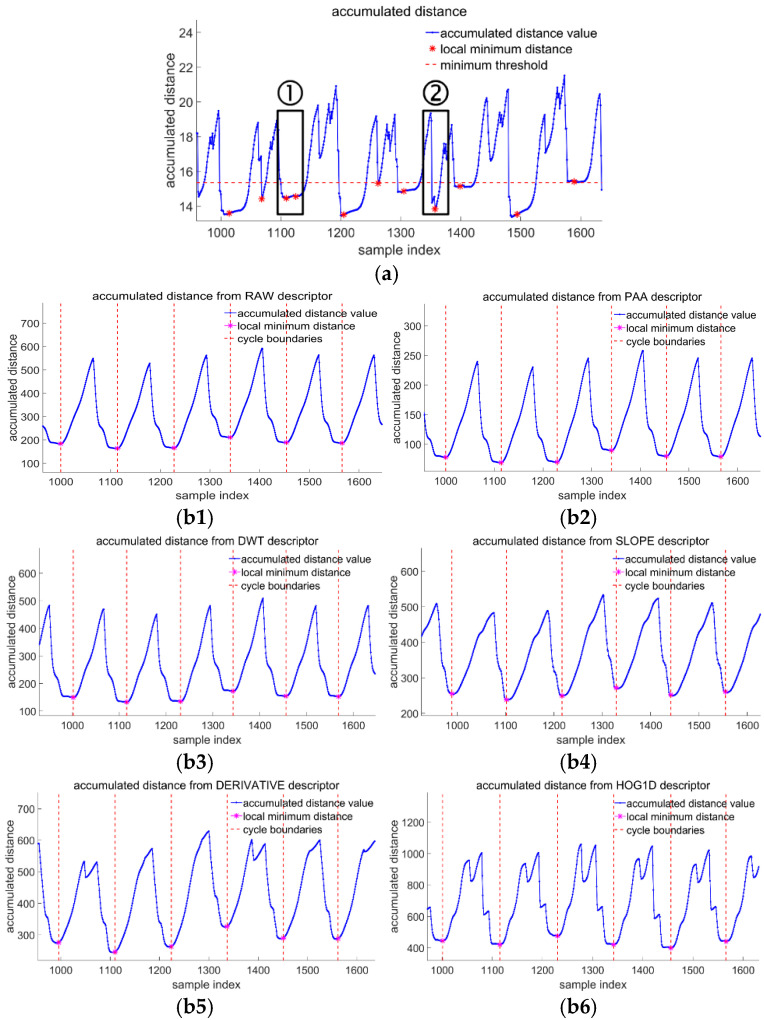
The accumulated distance curve used in conventional DTW methods may contain spikes leading to pseudo-minimums, and multi-valleys blurring the stride boundaries. Shape descriptors are able to improve the smoothness and monotonicity of the accumulated distance curve. (**a**) accumulated distance in conventional DTW; (**b1**) accumulated distance from RAW descriptor; (**b2**) accumulated distance from PAA descriptor; (**b3**) accumulated distance from DWT descriptor; (**b4**) accumulated distance from SLOPE descriptor; (**b5**) accumulated distance from DERIVATIVE descriptor; (**b6**) accumulated distance from HOG1D descriptor.

The $dist_{i,j}$ is the spatial distance between the i-th point in query sequency and the j-th element in template. It is usually calculated by using Euclidean distance, Manhattan Distance, Hamming Distance or other types of approach. The msDTW algorithm is one of the cases employing template matching algorithms to stride segmentation in recent years. In conventional DTW algorithms, the accumulated distance is compared with a threshold to assess whether a data segment fits the signal distribution in the template, which is also used in the msDTW method. Thus, msDTW can represent standard DTW algorithms that make decisions based on a threshold. We found that the distribution of accumulated distance is not monotonically increasing or decreasing when applying msDTW to data from the same subject with consistent walking cadence. A visible spike appears at the time adjacent to the valley point, as shown in box 2 in Figure 5. However, the threshold and temporal conditions fail to preclude the appearance of pseudo-valleys.

In this literature, the stride boundary is set at the end of the stance phase, which accounts for nearly 40% of a stride cycle. Because the magnitude of the sensor signal in the stance phase is lower than in other phases, the stride boundaries appear in the flat section of the accumulated distance curve. The phenomena of multiple valleys within the flat area make the basis for identifying the boundaries blurred, as indicated in box 1 in Figure 5. Therefore, if the curve of the accumulated distance function could keep smooth and monotonic, it would be promising to improve the accuracy of the stride segmentation method.

We use shape descriptors to help improve the smoothness and monotonicity of the accumulated distance curve. Shape descriptors were designed to measure similarities between two points by computing similarities between their local neighborhoods, rather than computing the distance between two points based on their values. As a result, the accumulated distance between the sample point and the template element is the difference between the data distribution of their neighborhoods and a subsequence of template, where the difference calculation is not confined to the Euclidean distance. By measuring the differences between neighbors, it makes the cumulative distance curve behave more smoothly and the valley spots more distinct and discriminative than conventional DTW approaches, which form the basis of SDATW without relying on thresholds. As shown in Figure 5, SDATW employs six shape descriptors, which aid the accumulated distance curve’s more desirable smoothness and monotonicity.

In the process of finding the minimum cumulative distance, the traditional DTW methods require recreating a sliding window for each sample point and calculating the distance between the data segment inside the window and the template, which makes the DTW computationally expensive. SDATW searches for the minimum accumulated distance point by using only a matrix to locate the data segment with starting and ending points that best match the template. The selected matching data segment can be guaranteed that its starting point has the best similarity to the template than the previous sample points, and the data before the ending point contain the information of the current cycle as much as possible, which allows SDATW to get rid of the dependence on the threshold. It ensures that the minimum value in the accumulated distance is not overlooked, as well as supports online stream data detection to suit practical application requirements. In this literature, we replace the spatial distance by measuring the distance between shape descriptors, which is designed to express subsequence structural information under the assumption that the optimal-matched subsequences should be recognized with the best similarity between their structural features.

Each shape descriptor is calculated to encode local structural information around the temporal point $\alpha_{i}$ or $\beta_{j}$. Given a mapping function M(⋅), we convert the query sequence A to its descriptor sequence: $A_{des}={\left\{\alpha_{des}{}_{0},\alpha_{des}{}_{1},\ldots ,\alpha_{des}{}_{m-1}\right\}}^{T},\alpha_{des}{}_{i}\in R^{l}\;$, i.e., $A_{des}={\left\{M\left(\alpha_{0}\right),M(\alpha_{1}),\ldots ,M(\alpha_{m-1})\right\}}^{T}$. The l in $\alpha_{des}{}_{i}\in R^{l}\;$ indicates that the dimension of $\alpha_{des}{}_{i}$ could be different with sample point and is up to the mapping function M(⋅). The shape descriptors can be classified into the magnitude-aware-descriptors and the fluctuation-capturing-descriptors. The magnitude-aware-descriptors include Raw Subsequence (RAW), Piecewise Aggregate Approximation (PAA), and Discrete Wavelet Transform (DWT). RAW is namely the raw samples around the point where features need to be extracted. PAA consists of mean values of several equal-length intervals divided from the original subsequence. DWT consists of concatenated wavelet coefficients, which come from decomposing a subsequence into three levels by using a Haar wavelet basis. These three kinds of descriptors are generated based on the amplitude of the signal, namely the y-axis value, so they memorize the signal magnitude distribution in the neighborhood of the sample point in the subsequence. The fluctuation-capturing-descriptors involve SLOPE, DERIVATIVE, and HOG1D. SLOPE is extracted as a series of gradients of the intervals whose length depends on the size of the subsequence and the number of equal-length intervals; DERIVATIVE is similar to SLOPE but is calculated from the first-order derivative of a subsequence; HOG1D inherits from the Histogram of Oriented Gradients (HOG) descriptor [50] and was used in [51] to describe 1D time series sequences. As SLOPE, DERIVATIVE, and HOG1D mainly record the direction and amplitude of signal fluctuations, they are invariant to the magnitude of raw subsequence. We refer interested readers to [52] for computation details of shape descriptors.

**Figure 6:**
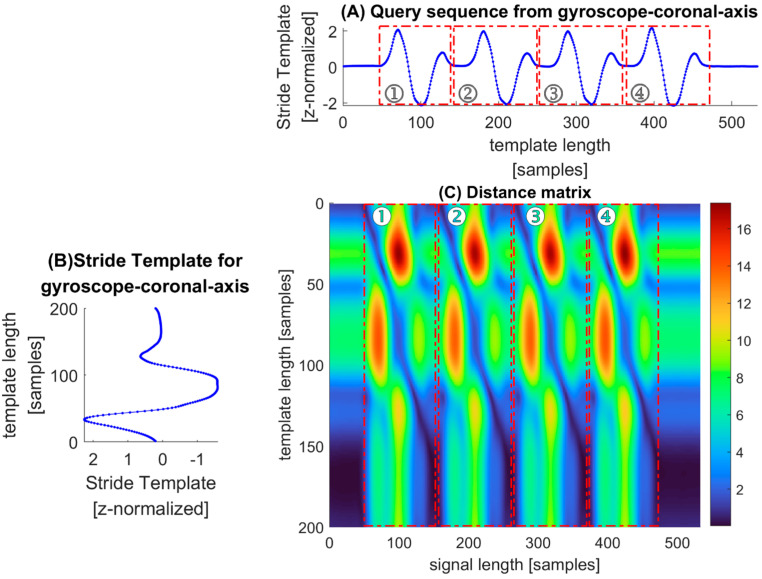
Distance matrix is shown as an example for calculating RAW descriptors for gyroscope-coronal-axis-data. The elements with deep blue in the distance matrix show closer spatial distance between the shape descriptor of a query sample and that of a template point, while elements with red indicate greater spatial distance. (**A**) query sequence $A_{des}$; (**B**) stride template $B_{des}$; (**C**) distance matrix dist;”

Figure 6 shows an example of calculating RAW descriptors for gyroscope-coronal-axis-data. Each column represents the distance between one sample of query sequence $A_{des}$ and the complete template $B_{des}$. The top row represents the distance between the beginning point of the template $B_{des}$ and the sequence $A_{des}$, while the bottom row represents the distance between the end of the template $B_{des}$ and the sequence $A_{des}$. It is clearly shown in Figure 6 that on the distance matrix, four dark blue paths are running from top to bottom through the matrix and featuring periodicity, which is same as the number of strides in IMU data. We would like to try to match each subsequence in the query sequence with the stride template, with the goal of minimizing the cumulative distance and finally finding the optimal segment.

By computing the weighted sum of one magnitude-aware-descriptor and one fluctuation-capturing-descriptor, we can get a compound shape descriptor, which may carry over the strengths of both descriptors and thus be more promising to correctly distinguish stride segment from IMU data streams.

#### 2.5.4. Augmented Time Warping Scheme

Given query sequence $A={\left\{\alpha_{0},\alpha_{1},\ldots ,\alpha_{m-1}\right\}}^{T},\;A\in R^{m}$ and template $B={\left\{\beta_{0},\beta_{1},\ldots ,\beta_{n-1}\right\}}^{T},\;B\in R^{n}$, let $A_{des}={\left\{\alpha_{des}{}_{0},\alpha_{des}{}_{1},\ldots ,\alpha_{des}{}_{m-1}\right\}}^{T},\alpha_{des}{}_{i}\in R^{l}$ and $B_{des}={\left\{\beta_{des}{}_{0},\beta_{des}{}_{1},\ldots ,\beta_{des}{}_{n-1}\right\}}^{T},\beta_{des}{}_{j}\in R^{l}$ be their shape descriptor sequences, respectively. Then, we use a disjoint query DTW method to find the $A_{des}{}^{\prime }={\left\{\alpha_{des}{}_{s},\alpha_{des}{}_{s+1},\ldots ,\alpha_{des}{}_{e}\right\}}^{T},\alpha_{des}{}_{i}\in R^{l}$ whose distance from template-descriptor sequence is the smallest among those of all other possible subsequences of $A_{des}$. That is to say, $D\left(A_{des}\left[s:e\right],\;B_{des}\;\right)\leq D\left(A_{des}\left[p:q\right],\;B_{des}\right)$ for any pair of p=0,1,…,m−1 and q=p,…,m−1. In this part of work, we combined the shape descriptors with the SPRING method proposed in [53]. Then, a threshold-independent DTW method was evaluated on the MATLAB platform for solving the stride segmentation problem. By augmenting the time warping process from two aspects, our method is able to find the best-match part from query subsequence but also dramatically reduce the computation complexity since it is invariant to the length of streaming data.

The calculation of accumulated distance of warping path is same as naïve solution, but a subsequence time warping matrix named $s_{query}$, whose height is same as the length of template B, is utilized to keep the beginning point of current candidate query in memory at the same time. The process of memorization is shown in following formula:

(5) $$s_{query}\left(t,\;i\right)=\{\begin{array}{cc} s_{query}\left(t,\;i-1\right), & D\left(t,i-1\right)==d_{best}\\ s_{query}\left(t-1,\;i\right), & D\left(t-1,\;i\right)==d_{best}\\ s_{query}\left(t-1,\;i-1\right), & D\left(t-1,\;i-1\right)==d_{best} \end{array}$$

(6) $$d_{best}=\min\left\{D\left(t,i-1\right),D\left(t-1,i\right),D\left(t-1,i-1\right)\right\}$$

In this direct way, the head of the current candidate query could be saved in a greedy way. In addition, if the candidate query is confirmed to be the best-match query at index-position t, the range of best match in query sequence will be readily available, the beginning position of $A_{des}\left[s:e\right]$ is the value saved in $s_{query}\left(e,\;end\right)$ and the end position is e. It should be noted that the confirmed position of the best match is later than the end position of the best match and the reason can be explained in the following section.

The guarantee of no false best match is a necessity for the success of this step. Even though pseudocode has been given in [53], we provide another perspective of understanding based on the version of the method we reproduced.

When a new point $\alpha_{des}{}_{t}$ from query sequence $A_{des}$ comes, we firstly calculate the accumulated distance results. This step of calculation actually generates a new column in the accumulated distance matrix D and a new column of beginning-position recordings in the time warping matrix $s_{query}\left(i,j\right)$. We use D(:,t) and $s_{query}\left(:,t\right)$ to represent the new columns, respectively, and the last element in D(:,t) and $s_{query}\left(:,t\right)$ is represented as D(end,t) and $s_{query}\left(end,t\right)$. The accumulated distance of current candidate query is named as optimal distance and represented as $d_{min}$.

We need to check whether current candidate query needs to be replaced. If judgement statement $D\left(end,t\right)<d_{min}$ is true, which means there is another waring path that matches the template with less differences, then the current candidate query should be replaced by the new query: let the optimal distance $d_{min}=$ D(end,t), the beginning position $s=s_{query}\left(end,t\right)$ and the end position e=t, and the index range of candidate query is [s:e]. It should be noted that each point that is located in the index range of a candidate query has the opportunity to act as a competitor to be the head of another candidate query, unless it is excluded from the competitors against the head. This step ensures that the best match will not be missed.

To beat and exclude the competitors which no longer have the chance to replace the current candidate query, we find the index position of the element in $s_{query}\left(:,t\right)$ which is competitor against the head. We use cmp to represent the index position of a competitor point. If the judgement statement $D\left(cmp,t\right)>d_{min},\;t<end$ is true, which replies the even the query matched with only part of template, its accumulated distance is larger than current candidate query, not to mention the accumulated distance of matching the whole template. In this way, the candidate query proves its superiority over its competitors. This step acts as the preparation of confirming the candidate query as best-match query.

To confirm the candidate query as a best-match query, we only need to make sure that there exist no competitor’s index positions in $s_{query}\left(:,t\right)$ or that there is hardly competitor’s accumulated distance that is smaller than $d_{min}$. These two circumstances both indicate that the candidate query stands out, since a new stride might have begun. The confirmed query will be saved in $opt_{query}.$ Here, we also provide the explanation for the confirmed position lagging behind the end point of the best match.

**Figure 7:**
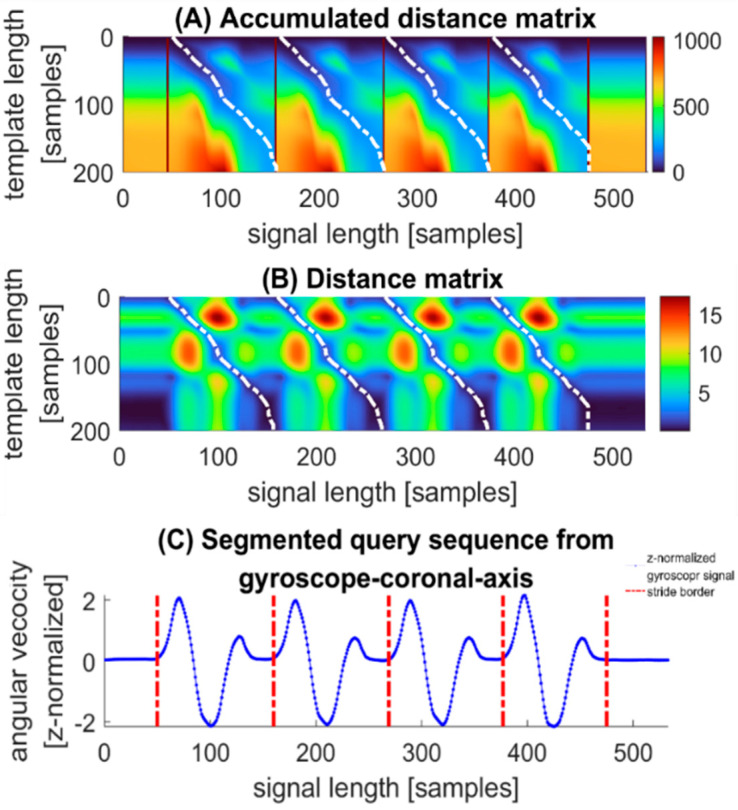
(**A**) The white lines represent the warping paths which correspond to best-matched subsequences in the query sequence. By using the augmented time warping scheme, the best-match subsequences could be detected and borders of strides recognized. Dark red ribbons between two warping paths indicate the borders of detected stride segments. They display the accumulated distances that are positive infinite resulting from star-padding. (**B**) The warping paths in distance matrix just run through the deep blue area from top to bottom, which is consistent with the hypothesis in Section 2.5.3. (**C**) After applying the time range of warping paths to the time axis of IMU data, the stride borders are available, which are represented as red vertical lines. Additionally, the stride segments just look like template.

The method to start looking for new best match is called “star-padding” [53]. We deliberately create a vector full of positive infinite elements with same height as D(:,t) and put it at the left side of D(:,t). As for $s_{query}\left(:,t\right)$, we create a vector full of zero and deal with it in the same way. The detected strides can be accessed by $opt_{query}$, which memorizes the time range of each detected stride. The results can be shown in Figure 7.

#### 2.5.5. Complexity Analysis

We make A an evolving sequence of length m, B a sequence of fixed length n, and l the size of each neighbor for extracting each shape descriptors.

According to [54], the calculation of shape-descriptors takes linear time O( l∗m). At time warping step, our algorithm keeps updating a single matrix and calculating O( n) numbers for each sample point, the time complexity of time warping is O( m∗n). Since l≪m and n≪m in general, the total time complexity of shape-descriptor calculation and time warping procedure are same as $O\left(\;m^{2}\right)$; hence, the time complexity of the whole algorithm is $O\left(\;m^{2}\right)$, which is the same as conventional DTW.

In summary, we prepare for warping path procedure with more in-depth information than conventional approach, including magnitude and fluctuation of the IMU signal. Furthermore, by recording the head of the candidate query and calculating the accumulated distance matrix simultaneously, the augmented time warping matrix makes it possible to calculate only one column of accumulated distance for each input point. Last but not least, the technique of searching for best-match queries allows us to avoid relying on thresholds and the procedure auto initializes when we detect a new stride segment. Because the algorithm is based on shape descriptors [54] and augmented time warping [53], it is named SDATW.

#### 2.5.6. Time Constraints

According to [8], the duration of a stride is located in the range of 250 to 2000 ms. This helps us to exclude the pseudo-stride whose time durations are not compatible with the time constraints.

### 2.6. Gait Division

When a pedestrian walks, successive gait patterns are sequentially articulated to form a complete stride, and the gait patterns are divided between them based on the foot movement. When the pushoff phase ends, the toe leaves the ground and the swing phase begins; when the swing phase ends, the heel makes contact with the ground and the heel-strike phase begins; when the heel-strike phase ends, the palm of the foot (shoe) makes full contact with the ground and the stationary phase begins; when the stationary phase ends, the heel begins to leave the ground and the pushoff phase begins.

**Figure 8:**
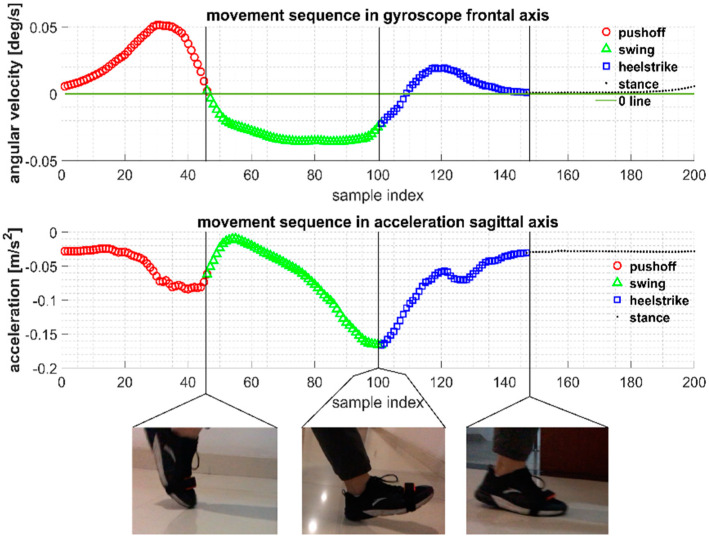
The time range of gait labels in video recordings can be converted to IMU sequence with the assurance of time synchronization, which performs as the basis of gait analysis and gait phase recognition.

Therefore, we define Toe Off (TO), Heel Touch (HT), and Heel Off (HO) as three different stride events for gait recognition. Based on the time synchronization between IMU data and gait label, we can apply the time range of gait labels to IMU sequence, and experience-based knowledge of gait borders was verified to be a gait recognition method [10]. When a TO event occurs, the foot completes the action from plantar flexion to dorsiflexion, which leads to the gyroscope detecting a zero crossing of the coronal axis data; when a HT event occurs, the gyroscope data in the coronal axis direction is reflected to a sharp change of data in one direction due to the violent interaction between the foot and the ground, and the accelerometer in the sagittal axis direction is reflected to a spike of signal due to the sudden change of force; when an HO event occurs, which breaks the static phase of the foot, the detection is performed by setting a threshold for the variance of the signal, from which the division of the stride and gait is obtained, as shown in Figure 8.

### 2.7. Error Measurement

There are two types of errors in stride segmentation and gait recognition: (1) stride segmentation failed to recognize the labeled stride boundaries; (2) stride segmentation is excessively sensitive and detects pseudo-stride boundaries. These types of errors also occur in gait division assignments. Therefore, a preliminary evaluation of stride segmentation and gait recognition based on accuracy and recall is performed.

#### 2.7.1. Precision


(7) $$precision=\frac{\sum true\;positives}{\sum detected\;positives}$$

The true positives means the number of detected strides that are true in labels. The detected positives is the number of all strides detected by the algorithm. *Precision* is used to measure the level of correct detection of strides and gaits by the algorithm.

#### 2.7.2. Recall


(8) $$recall=\frac{\sum true\;positives}{\sum true\;positives+\sum false\;negatives}$$

The *recall* is the proportion of detected true strides/true gaits to all paces/gaits that are labeled. All labeled strides/gaits contain samples that may not be detected by the algorithm and are therefore used to measure the level of failure of the algorithm to detect the labeled strides/gaits.

#### 2.7.3. F-Measure


(9) $$F-measure=2\cdot \frac{precision\cdot recall}{precision+recall}$$

The F–measure is the summed average of the accuracy and *recall* [22] and contains the strides/gaits that were falsely detected as well as those that were not detected.

## 3. Experiments and Results

To evaluate the effectiveness of SDATW, we carried out experiments on the diverse gait dataset in four parts. Firstly, we tested the stride segmentation performance of six single-axis schemes based on the magnitude-aware-descriptors and picked the best one; then we selected a kind of descriptor from the fluctuation-capturing-descriptors that works best in the same way. Secondly, based on the experience of the first part, we tried axis combinations for the selected shape descriptors and performed stride segmentation. In the third part, we fused the selected shape descriptors to form a compound shape descriptor and tried all single-axis and multi-axis combinations. In the fourth part, we presented the results of gait recognition based on the optimal scheme of stride segmentation. Our final goal is to try to find the best decision of descriptor-data-axis schemes and use this result as a benchmark for the diverse gait dataset. The IMU module being worn loosely during the walking process causes the acceleration and gyroscope data to become noisier and alters the distribution pattern of a stride cycle. In order to mitigate the detrimental impacts on stride segmentation, we adopted a simple strategy: both the original and its flipped version were employed as the inputs, and the group with higher number of identified strides was chosen as the formal output of SDATW.

### 3.1. Separate Performance Evaluation of Two Types of Shape Descriptors

The goal of this portion of the experiment is to use each single kind of shape descriptor to implement and validate pedestrian stride segmentation under cross-individual and broad speed domain circumstances. Therefore, when preparing the experimental data, we presume that the algorithm should not have access to certain priori information aids, such as the physiological features of the pedestrians. According to gait analysis in [55], walking speed dominates in influencing gait parameters over gender, age, body height and weight. Therefore, we consider walking speed type as the key component in the test scenario in order to verify the stability of our stride segmentation method. For each group of speed-type test, we mixed foot movement data from all subjects, which brought the diversity of genders and height groups.

The SDATW was implemented for each distinct axis of acceleration and gyroscope. Thanks to the contribution of [54], we could try various shape descriptors and test their capability in extracting signal characteristics. Table 3 and Table 4 present an overview of the performance using the magnitude-aware-descriptors and the fluctuation-capturing-descriptors, respectively.

Firstly, by comparing the errors of different speed groups, we can identify whether the performance of the stride division algorithm based on one shape descriptor is stable under different walking speeds. Secondly, we can also determine the IMU data axes that are most significant to the stride segmentation by comparing the results with different data axes. Finally, the data from all velocity groups are combined together to get the average accuracy of the stride segmentation across individuals and wide velocity domain scenarios. We could settle on the proper sensor axes and the shape descriptors that stand out with the highest average F-measure. In experiments using the magnitude-aware-descriptors for stride segmentation, the best performance could be obtained by using coronal gyroscope axis data and selecting DTW or PAA as shape descriptors. In fluctuation-capturing-descriptors, HOG1D based on vertical gyroscope data was selected as the best one.

### 3.2. Stride Segmentation with Selected Shape Descriptors and Combined Sensor Types

Based on DWT, PAA, and HOG1D, we used the combination of accelerometer and gyroscope data to see if there was an improvement in the results. Table 5 presents an overview of the performance using different sensor axis combination schemes. The best choice for DWT and PAA is using acceleration along the sagittal axis and the vertical axis together and the best choice for the sensor axis combination is using three-axis-gyroscope data together, but we need to say that there is a significant lag compared to the best result in first part of experiments.

### 3.3. Stride Segmentation with Compound Shape Descriptors

We concatenated HOG1D as a fluctuation-capturing-descriptor and all three magnitude-award-descriptors with equal weights, resulting in three compound descriptors: HOG1D+RAW=(HOG1D,RAW), HOG1D+DWT=(HOG1D,DWT), HOG1D+PAA=(HOG1D,PAA).

Then, we evaluated stride segmentation with all sensor axis plans. The results are showing in Table 6. We discovered that the best combination of magnitude-aware-descriptors (*RAW*, *PAA*, and *DWT*) with *HOG*1*D* is *RAW* + *HOG*1*D*, rather than *DWT* + *HOG*1*D*, in which *DWT* owned the best performance among magnitude-aware-descriptors. This result confirms the idea that combining the magnitude-aware-shape descriptors and the fluctuation-capturing-descriptors might boost algorithm performance.

We compare SDATW with two stride segmentation methods commonly used in current wearable devices. msDTW uses a grid search algorithm to optimize the thresholds while fusing data from multiple sensor data axes, thus ensuring both the accuracy of stride detection and good cross-individual and wide speed domain adaptability. The wavelet-based algorithm extracts features of the signal based on multi-scale analysis, so the thresholds in the algorithm also have good adaptability to different pedestrian individuals and different walking speeds [45]. The comparison results for stride segmentation on the diverse gait dataset is shown in Table 7. According to [8], grid search method must be applied to find the optimal threshold for each of the different speed groups, so it is reasonable that msDTW performs slightly better than the best result of our algorithm in fast and slow speed tests. However, when applied in real scenarios, our method could maintain accuracy and robustness with no preparation while msDTW might not. Compared with the wavelet-based method, SDATW generally performed slightly better, except in the slow speed test.

### 3.4. Gait Recognition with Optimal Shape Descriptor and Sensor Type

In this part of the work, we evaluated the gait division based on the stride segmentation of the optimal scheme. The results in Table 8 show the effect of walking speed on the gait recognition. The boundaries between gait patterns are blurred due to the relatively weak amplitude of foot movements during slow walking, so F-measures in the slow group are smaller than those in other groups.

## 4. Discussion

In a PDR system, the pedestrian’s position could be updated by

(10) $$P_{t}=\left[\begin{array}{c} P_{t}^{N}\\ P_{t}^{E} \end{array}\right]=\underset{P_{t-1}}{\underset{︸}{\left[\begin{array}{c} P_{t-1}^{N}\\ P_{t-1}^{E} \end{array}\right]}}+SL_{t}\left[\begin{array}{c} \cos\left(\phi_{t}\right)\\ \sin\left(\varphi_{t}\right) \end{array}\right]$$

where $P_{t}$ and $P_{t-1}$ are the current and latest positions, respectively; $P_{t}^{N}$ and $P_{t}^{E}$ are the displacements in the north and east directions of PDR’s coordinate system, respectively. Based on the known stride boundaries, we may divide a stride into four gait phases based on the stride boundaries: stance, pushoff, swing, and heel-strike phases. From the perspective of gait analysis, the total of the foot-moving distances of the pushoff, swing, and heel-strike phases, also known as dynamic gait phases, determines the walking distance of the foot in a stride time range. It is also necessary to estimate the direction of foot motion during the stride time based on IMU data and magnetometer data. This problem is outside the scope of this paper, but we found that there has been extensive research in the field of localization for direction estimation based on IMU data [6,15,56,57,58,59]. The SDATW proposed in this literature is expected to produce accurate stride segmentation and strong robustness in cross-individual and wide speed domain walking scenarios due to its threshold independence and adaptability.

For the choice of shape descriptors, we employed two kinds of shape descriptors for stride segmentation; however, it was found that the fluctuation-capturing-descriptors did not work well as the magnitude-aware-descriptors. We infer that this result stems from their properties. Our template is generated by randomly selecting 30% of the strides from each speed group. This process is itself a smoothing of the stride signal, leading to a smoother segment to stand for a typical stride signal for each axis of IMU data. However, the fluctuation-capturing-descriptors (SLOPE, DERIVATIVE, HOG1D) essentially record the fluctuations of the input signal, while the fluctuations in the template signal have already been weakened, which makes them weaker than magnitude-aware-descriptors in matching the template both on acceleration data and gyroscope data.

Among SLOPE and DERIVATIVE, each item is a gradient obtained by linear regression on a small set consisting of sample points and their neighbors. However, linear regression is easily subject to outliers, whereas HOG1D obtains a histogram of the slope distribution-oriented gradient (HOG) descriptor [60], which means that HOG1D retains more knowledge about the slope than linear regression and could be more tolerant to outliers.

In the comparison of all the axes in the IMU data, the coronal axis gyroscope data tend to exhibit the closest periodicity and smoothness to the template, and we infer from two perspectives. Firstly, when a pedestrian is walking, the coronal axis in the gyroscope records the angular velocity of rotation of the ankle as it transitions back and forth from plantar flexion to dorsiflexion. The motion pattern of the ankle is quite homogeneous under walking state, so the signal amplitude of the coronal axis gyroscope is greater than that of the sagittal and vertical axes. This phenomenon is most obvious when a pedestrian is walking in a straight line. Even if the pedestrian makes a turn or other movement, plantar extension and dorsal extension of the ankle are still the keys to generating driving force in the lower limb. Thus, the gyroscope signal of the coronal axis should be considered as the most essential motor information in stride segmentation and gait recognition with respect to the other two axes. Furthermore, the gyroscope records the effect of the force driving the rotation accumulated per unit time, while the accelerometer records the immediate effect of the force acting on the object. Consequently, the acceleration signal during walking has more burrs than the gyroscope signal. Additionally, the variation in walking habits between individuals further increases the difference between the actual acceleration pattern and the pattern within the template. We think it is harder to detect strides based on acceleration structural features compared to that of the gyroscope. Although we use mean filtering to smooth the acceleration signal, we still cannot get the acceleration stream to be as ideal as shown in the template. If the size of the sliding window is large to make burrs disappear, it destroys valuable information in the original signal, which will not be compensated by any subsequent step, such as z-normalization or extracting shape descriptors.

We have tried all combinations of accelerations to try to improve the performance of the acceleration-based shape descriptors for stride segmentation, but it still performed similarly to the single-axis-data. Here, we think that the acceleration data fusion method needs to be modified. The axis combination method in our algorithm is the calculation of the modulus of two-axis data or three-axis data, which is essentially calculating the 2-norm fusion of data from different axes. However, the signal along different axes records three mutually orthogonal motion components of foot motion in space, and their noise distributions are likely to be uncorrelated. With the implementation of axis fusion, the noise along all axes is not eliminated, but is retained. This might bring more noise information to shape descriptors rather than highlighting the periodic patterns of the signal.

We agree that if the tri-axis acceleration data are properly utilized and fused with gyroscope data, it will further improve the robustness of the stride segmentation algorithm in different individuals and under complex scenarios [8,35]. Therefore, in our future work, we will investigate and explore IMU data fusion methods to improve the accuracy and generalizability of stride segmentation. In addition, for the problem of pedestrian step length estimation based on gait analysis, we have only implemented and validated it for one subject under a free-walking scenario. By utilizing reference datasets for pedestrian navigation as a testbed, we are going to study an adaptive step length estimation model on the basis of gait analysis that is capable of providing distance prediction with high accuracy and proficient adaptability for different individuals.

## 5. Conclusions

This paper provides a diverse gait dataset with comprehensive coverage of healthy subjects by gender, height, and walking speed. There are 4690 strides of walking data collected and 19,083 items annotated as gait labels. Furthermore, based on this dataset, a novel algorithm called SDATW was proposed in the literature. With no dependence on the threshold, the SDATW algorithm could be used for stride segmentation with no customization for individual pedestrians and can also maintain accuracy under different walking speeds. The best F-measure for fast walking, normal walking and slow walking is 0.813, 0.818 and 0.829, respectively. The performance of SDATW is slightly better than that of the conventional DTW algorithm and wavelet-based method, with no parameter optimization process. Last but not least, a gait recognition method was evaluated on the basis of SDATW’s output, and the detailed results could be used as the baseline for gait recognition on the diverse gait dataset.

## Figures and Tables

**Table 1:** Summary of published gait datasets.

Dataset	Digital Biobank		Sensor-Based Gait Analysis Validation Data [30]	MAREA [31]	Smart Annotation Cyclic Activities Dataset [32]	The Diverse Gait Dataset
	eGaIT-Validation Stride Segmentation [8]	eGaIT-Validation Gait Parameters [10]				
Sampling frequency [Hz]	102.4	102.4	102.4	128	200	100
Reference Data	GAITRite (pressure sensors)	Manual annotation	Motion capture system	Piezo-electric force sensitive resistors	Camera recordings (30 Hz)	Camera recordings
Number of subjects	101 (55 females and 46 males)	70 (39 males and 41 females)	15 (8 males and 7 females)	20 (12 males and 8 females)	18 (14 males and 4 females)	22 (13 males and 9 females)
Subject health description	Generic patients.	Elderly controls (45), PD patients (15), geriatric patients (15).	Healthy (11), PD patients (4).	All healthy.	All healthy.	All healthy.
Scenarios	laboratory settings	Indoor: obstacle-free environment; Outdoor: overground.	Laboratory settings.	Indoor: laboratory settings; Outdoor: overground streets.	Outdoor: A prescribed circuit in outdoor setting with varying surfaces.	Indoor corridors.
Sensor wear positions	Shoe	Shoe	Shoe	Waists, left wrist, left and right ankles	Shoe	Shoe
Labels	Gait velocity, cadence, step length, heel to heel base of support width, length of gait phases. [33]	The start and end point of each stride	Heel-strike, toe-off, heel-off	Heel-strike, toe-off	The start and end point of each stride	Stance, toe-off, heel-strike.
Walking distance/duration	10 m normal walk; 1–2 min four-wheeled walk;	40 m straight walk; 2 min free walk;	4 × 10 m walk;	Treadmill walk; outdoor walk/run/jog;	-	46 m straight walk
Number of strides	-	-	1116 strides (1037 from healthy subjects, 129 from patients.)	-	2263 walking strides and 1391 running strides	4690 walking strides

**Table 2:** A total of 22 healthy volunteers (13 males, 9 females, age 32.5 ± 7.5 years) participated in the study and were divided into different groups according to gender and height information.

Height Range (cm)	Males	Females	Number of Strides (Speed Type)		Number of Gait Phases	
155~160	-	2	fast	142	stance	487
			middle	159	pushoff	478
			slow	171	swing	474
			all	472	heel-strike	474
160~165	2	3	fast	261	stance	1121
			middle	337	pushoff	1100
			slow	475	swing	1113
			all	1073	heel-strike	1110
165~170	2	2	fast	298	stance	1022
			middle	324	pushoff	1013
			slow	367	swing	1113
			all	989	heel-strike	998
170~176	4	1	fast	406	stance	1358
			middle	440	pushoff	1353
			slow	459	swing	1006
			all	1305	heel-strike	1378
176~180	2	1	fast	123	stance	408
			middle	121	pushoff	399
			slow	146	swing	400
			all	390	heel-strike	393
180~185	3	-	fast	150	stance	480
			middle	114	pushoff	470
			slow	197	swing	470
			all	461	heel-strike	465

**Table 3:** Stride segmentation results for magnitude-aware-descriptors in F-measure values. Best results for each speed group are highlighted in bold numbers.

Shape Dscriptor	Speed	AccX	AccY	AccZ	GyroX	GyroY	GyroZ
RAW	fast	0.623 ± 0.022	0.499 ± 0.048	0.537 ± 0.056	0.328 ± 0.049	0.605 ± 0.018	0.737 ± 0.069
	mid	0.505 ± 0.066	0.681 ± 0.042	0.559 ± 0.088	0.362 ± 0.051	0.727 ± 0.049	0.832 ± 0.019
	slow	0.534 ± 0.077	0.807 ± 0.012	0.607 ± 0.067	0.449 ± 0.069	0.774 ± 0.029	0.831 ± 0.008
	all	0.63 ± 0.052	0.654 ± 0.043	0.577 ± 0.072	0.426 ± 0.07	0.675 ± 0.063	0.796 ± 0.034
PAA	fast	0.758 ± 0.015	0.65 ± 0.071	0.306 ± 0.055	0.256 ± 0.042	0.642 ± 0.024	**0.819 ± 0.029**
	mid	0.783 ± 0.025	0.748 ± 0.057	0.35 ± 0.066	0.232 ± 0.061	0.73 ± 0.038	**0.852 ± 0.006**
	slow	0.691 ± 0.033	0.76 ± 0.024	0.377 ± 0.04	0.477 ± 0.071	0.769 ± 0.033	**0.816 ± 0.011**
	all	0.784 ± 0.019	0.666 ± 0.06	0.265 ± 0.043	0.278 ± 0.052	0.705 ± 0.057	**0.833 ± 0.013**
DWT	fast	0.765 ± 0.012	0.451 ± 0.081	0.304 ± 0.051	0.294 ± 0.052	0.652 ± 0.031	**0.811 ± 0.031**
	mid	0.782 ± 0.027	0.638 ± 0.051	0.333 ± 0.06	0.206 ± 0.038	0.67 ± 0.025	**0.847 ± 0.007**
	slow	0.739 ± 0.029	0.687 ± 0.05	0.375 ± 0.056	0.38 ± 0.069	0.722 ± 0.029	**0.806 ± 0.011**
	all	0.761 ± 0.026	0.497 ± 0.07	0.243 ± 0.045	0.22 ± 0.051	0.687 ± 0.042	**0.835 ± 0.008**

**Table 4:** Stride segmentation results for fluctuation-capturing-descriptors in F-measure values. Best results for each speed group are highlighted in bold numbers.

Shape Dscriptor	Speed	AccX	AccY	AccZ	GyroX	GyroY	GyroZ
SLOPE	fast	0.016 ± 0.001	0.014 ± 0.001	0.015 ± 0.001	0.025 ± 0.001	0.109 ± 0.043	0.059 ± 0.023
	mid	0.032 ± 0.007	0.045 ± 0.008	0.014 ± 0.001	0.046 ± 0.005	0.165 ± 0.043	0.207 ± 0.072
	slow	0.148 ± 0.042	0.203 ± 0.037	0.115 ± 0.025	0.296 ± 0.066	0.44 ± 0.059	0.414 ± 0.115
	all	0.071 ± 0.02	0.069 ± 0.015	0.029 ± 0.004	0.146 ± 0.052	0.157 ± 0.039	0.245 ± 0.093
DERIVATIVE	fast	0.014 ± 0	0.019 ± 0.001	0.013 ± 0.001	0.023 ± 0.001	0.112 ± 0.049	0.067 ± 0.025
	mid	0.026 ± 0.003	0.041 ± 0.004	0.016 ± 0.001	0.041 ± 0.003	0.161 ± 0.044	0.249 ± 0.083
	slow	0.198 ± 0.063	0.293 ± 0.079	0.157 ± 0.04	0.304 ± 0.067	0.447 ± 0.075	0.418 ± 0.124
	all	0.109 ± 0.04	0.111 ± 0.039	0.052 ± 0.015	0.145 ± 0.05	0.167 ± 0.049	0.261 ± 0.1
HOG1D	fast	0.13 ± 0.031	0.228 ± 0.073	0.237 ± 0.071	0.179 ± 0.026	**0.578 ± 0.052**	0.094 ± 0.025
	mid	0.154 ± 0.021	0.455 ± 0.091	0.246 ± 0.058	0.166 ± 0.03	**0.639 ± 0.036**	0.218 ± 0.048
	slow	0.238 ± 0.047	0.232 ± 0.059	0.273 ± 0.061	0.308 ± 0.039	**0.65 ± 0.027**	0.504 ± 0.054
	all	0.186 ± 0.04	0.257 ± 0.065	0.285 ± 0.056	0.267 ± 0.054	**0.454 ± 0.07**	0.334 ± 0.078

**Table 5:** Stride segmentation results of different sensor axis combination schemes in F-measure values. Best results for each speed group are highlighted in bold numbers.

Shape Dscriptor	Speed	AccXY	AccXZ	AccYZ	AccXYZ	GyroXY	GyroXZ	GyroYZ	GyroXYZ
DWT	fast	0.585 ± 0.015	0.607 ± 0.03	**0.649 ± 0.043**	0.596 ± 0.009	0.504 ± 0.042	0.251 ± 0.083	0.273 ± 0.083	0.304 ± 0.082
	mid	0.593 ± 0.017	0.523 ± 0.07	**0.682 ± 0.023**	0.589 ± 0.018	0.305 ± 0.054	0.375 ± 0.067	0.381 ± 0.081	0.386 ± 0.058
	slow	0.669 ± 0.03	0.543 ± 0.063	**0.767 ± 0.015**	0.674 ± 0.032	0.487 ± 0.046	0.742 ± 0.053	0.718 ± 0.055	0.699 ± 0.061
	all	0.628 ± 0.019	0.581 ± 0.072	**0.666 ± 0.033**	0.624 ± 0.018	0.388 ± 0.065	0.347 ± 0.108	0.336 ± 0.098	0.337 ± 0.102
PAA	fast	0.512 ± 0.035	0.592 ± 0.045	**0.652 ± 0.058**	0.516 ± 0.044	0.364 ± 0.066	0.144 ± 0.04	0.122 ± 0.022	0.076 ± 0.013
	mid	0.552 ± 0.043	0.432 ± 0.051	**0.723 ± 0.038**	0.561 ± 0.038	0.219 ± 0.047	0.421 ± 0.066	0.385 ± 0.075	0.393 ± 0.074
	slow	0.659 ± 0.031	0.494 ± 0.063	**0.778 ± 0.017**	0.654 ± 0.048	0.4 ± 0.053	0.794 ± 0.051	0.813 ± 0.047	0.805 ± 0.041
	all	0.602 ± 0.032	0.557 ± 0.068	**0.697 ± 0.034**	0.602 ± 0.034	0.348 ± 0.061	0.441 ± 0.128	0.41 ± 0.121	0.405 ± 0.128
HOG1D	fast	0.628 ± 0.023	0.274 ± 0.041	0.639 ± 0.029	0.609 ± 0.017	0.654 ± 0.009	0.567 ± 0.076	0.632 ± 0.066	**0.571 ± 0.055**
	mid	0.603 ± 0.033	0.319 ± 0.037	0.59 ± 0.027	0.598 ± 0.024	0.74 ± 0.022	0.649 ± 0.012	0.677 ± 0.019	**0.662 ± 0.021**
	slow	0.638 ± 0.045	0.544 ± 0.037	0.674 ± 0.042	0.63 ± 0.042	0.735 ± 0.024	0.724 ± 0.03	0.745 ± 0.014	**0.724 ± 0.026**
	all	0.538 ± 0.042	0.313 ± 0.054	0.576 ± 0.042	0.552 ± 0.043	0.642 ± 0.028	0.759 ± 0.039	0.776 ± 0.034	**0.782 ± 0.033**

**Table 6:** Stride segmentation results of compound descriptor of different sensor axis combination schemes in F-measure values. Best results for each speed group are highlighted in bold numbers.

Shape Dscriptor	Single Axis	AccX	AccY	AccZ		GyroX	GyroX	GyroZ	
(HOG1D, RAW)	fast	0.515 ± 0.028	0.584 ± 0.031	0.325 ± 0.064		0.339 ± 0.046	0.566 ± 0.022	**0.815 ± 0.021**	
	mid	0.409 ± 0.067	0.721 ± 0.008	0.28 ± 0.071		0.305 ± 0.041	0.676 ± 0.043	**0.83 ± 0.021**	
	slow	0.438 ± 0.068	0.835 ± 0.008	0.253 ± 0.063		0.438 ± 0.068	0.732 ± 0.029	**0.824 ± 0.009**	
	all	0.427 ± 0.061	0.641 ± 0.034	0.276 ± 0.057		0.311 ± 0.052	0.636 ± 0.072	**0.823 ± 0.015**	
(HOG1D, DWT)	fast	0.443 ± 0.05	0.557 ± 0.039	0.301 ± 0.069		0.361 ± 0.043	0.599 ± 0.011	0.771 ± 0.038	
	mid	0.317 ± 0.051	0.703 ± 0.027	0.259 ± 0.056		0.36 ± 0.037	0.629 ± 0.038	0.798 ± 0.021	
	slow	0.399 ± 0.051	0.747 ± 0.034	0.267 ± 0.043		0.423 ± 0.04	0.702 ± 0.032	0.788 ± 0.012	
	all	0.372 ± 0.061	0.598 ± 0.038	0.3 ± 0.056		0.312 ± 0.054	0.625 ± 0.059	0.795 ± 0.017	
(HOG1D, PAA)	fast	0.295 ± 0.048	0.647 ± 0.04	0.355 ± 0.072		0.352 ± 0.061	0.598 ± 0.014	0.758 ± 0.059	
	mid	0.307 ± 0.059	0.765 ± 0.015	0.334 ± 0.075		0.404 ± 0.048	0.68 ± 0.038	0.817 ± 0.022	
	slow	0.413 ± 0.062	0.758 ± 0.026	0.206 ± 0.039		0.398 ± 0.041	0.744 ± 0.019	0.741 ± 0.03	
	all	0.291 ± 0.056	0.623 ± 0.041	0.299 ± 0.067		0.311 ± 0.056	0.652 ± 0.058	0.776 ± 0.033	
	Fuse axis	AccXY	AccXZ	AccYZ	AccXYZ	GyroXY	GyroXZ	GyroYZ	GyroXYZ
(HOG1D, RAW)	fast	0.583 ± 0.031	0.454 ± 0.05	0.612 ± 0.019	0.572 ± 0.023	0.533 ± 0.03	0.175 ± 0.039	0.352 ± 0.076	0.188 ± 0.049
	mid	0.546 ± 0.037	0.389 ± 0.058	0.67 ± 0.034	0.542 ± 0.041	0.616 ± 0.033	0.528 ± 0.092	0.609 ± 0.083	0.584 ± 0.097
	slow	0.631 ± 0.03	0.424 ± 0.065	0.796 ± 0.021	0.653 ± 0.017	0.626 ± 0.038	0.799 ± 0.032	0.837 ± 0.015	0.803 ± 0.032
	all	0.59 ± 0.048	0.38 ± 0.068	0.619 ± 0.035	0.571 ± 0.052	0.521 ± 0.054	0.399 ± 0.113	0.409 ± 0.114	0.385 ± 0.116
(HOG1D, DWT)	fast	0.574 ± 0.033	0.386 ± 0.062	0.572 ± 0.027	0.562 ± 0.033	0.538 ± 0.035	0.206 ± 0.038	0.341 ± 0.061	0.259 ± 0.056
	mid	0.518 ± 0.036	0.359 ± 0.058	0.516 ± 0.039	0.529 ± 0.042	0.626 ± 0.023	0.49 ± 0.043	0.494 ± 0.061	0.511 ± 0.04
	slow	0.598 ± 0.038	0.366 ± 0.066	0.72 ± 0.031	0.596 ± 0.036	0.626 ± 0.042	0.72 ± 0.04	0.776 ± 0.026	0.766 ± 0.027
	all	0.518 ± 0.061	0.319 ± 0.057	0.565 ± 0.043	0.512 ± 0.058	0.55 ± 0.052	0.465 ± 0.087	0.434 ± 0.098	0.416 ± 0.092
(HOG1D, PAA)	fast	0.564 ± 0.023	0.315 ± 0.054	0.63 ± 0.017	0.529 ± 0.024	0.536 ± 0.029	0.262 ± 0.059	0.34 ± 0.061	0.301 ± 0.058
	mid	0.496 ± 0.046	0.349 ± 0.067	0.567 ± 0.051	0.512 ± 0.04	0.707 ± 0.02	0.447 ± 0.051	0.536 ± 0.034	0.45 ± 0.055
	slow	0.515 ± 0.051	0.379 ± 0.055	0.764 ± 0.016	0.492 ± 0.046	0.714 ± 0.022	0.721 ± 0.036	0.781 ± 0.02	0.756 ± 0.029
	all	0.46 ± 0.059	0.287 ± 0.054	0.616 ± 0.03	0.468 ± 0.054	0.585 ± 0.034	0.436 ± 0.093	0.503 ± 0.085	0.508 ± 0.094

**Table 7:** Stride segmentation results of msDTW, wavelet-based method and SDATW in F-measure.

	msDTW	Wavelet Based Method	SDATW
fast	0.813	0.714	0.811
mid	0.818	0.781	0.847
slow	0.829	0.815	0.806
all	0.822	0.773	0.835

**Table 8:** Detailed results of gait phase recognition with different walking speed types given in F-measure values.

Walking Speed	Stance	Pushoff	Swing	Heel-Strike
Fast	0.8046	0.8522	0.8596	0.7884
Middle	0.7784	0.8461	0.8835	0.8056
Slow	0.701	0.6958	0.8399	0.7180
Full Range	0.7548	0.7925	0.8597	0.7674
